# Does helminthic infection alter the clinical course of HTLV-1 infection?

**DOI:** 10.1186/1742-4690-8-S1-A47

**Published:** 2011-06-06

**Authors:** Michael A Sundberg, Marshall J Glesby, Edgar M Carvalho

**Affiliations:** 1 Stanford University School of Medicine, Stanford, CA, 94305, USA; 2 Weill Cornell Medical College, New York, NY, 10065, USA; 3 Serviço de Imunologia, Hospital Universitário Professor Edgard Santos, Universidade Federal da Bahia, Salvador, BA, Brazil

## Introduction

HTLV-1 is associated with the development of HAM/TSP and overactive bladder (OB). A higher prevalence of helminthic coinfection has been observed among those infected with HTLV-1. Because helminthic infection may modify the immune response and influence clinical outcomes in HTLV-1 infection, we investigated the development of HAM/TSP and OB in HTLV-1 positive individuals with and without helminthic coinfection.

## Methods

HTLV-1 patients enrolled in a cohort study between 2004-2010 were classified as coinfected and non-coinfected based on stool samples. All patients were followed at least two years from initial evaluation, with yearly clinical assessments. Disease-free survival for OB was estimated using the Kaplan-Meier method, and the relationship between helminthic infection and development of OB was assessed by Cox proportional hazard modeling.

## Results

Seventy-four coinfected and 79 non-coinfected patients were followed. A total of 92 helminthic infections were observed in the coinfected group. One patient from each group developed HAM/TSP during followup. Fourteen and 17 patients developed OB from the coinfected and non-coinfected groups, respectively. There was no association between helminthic infection and risk of OB (hazard ratio 0.91, 95% CI 0.43-1.89, p = 0.79, adjusted for sex).

## Discussion

We found no difference in the risk of development of OB in HTLV-1 and helminthic coinfected and non-coinfected patients. The incidence of HAM/TSP was low in each group. These data indicate that general helminthic infection does not modify development of HAM/TSP or OB. Future studies should address the potential association between specific helminthic infections and risk of neurologic disease in HTLV-1 infection.

